# Decreased miR-132 plays a crucial role in diabetic encephalopathy by regulating the GSK-3β/Tau pathway

**DOI:** 10.18632/aging.202418

**Published:** 2020-12-27

**Authors:** Li Shi, Rui Zhang, Tian Li, Xue Han, Nannan Yuan, Lei Jiang, Huimin Zhou, Shunjiang Xu

**Affiliations:** 1 Department of Endocrinology, The First Hospital of Hebei Medical University, Shijiazhuang 050000, China; 2 Central Laboratory, The First Hospital of Hebei Medical University, Shijiazhuang 050000, China; 3 Department of Endocrinology, The First Affiliated Hospital of Hebei North University, Zhangjiakou 075000, China; 4 School of Basic Medicine, The Fourth Military Medical University, Xi'an 710032, China; 5 Department of General Practice, Xingtai People’s Hospital, Xingtai 054000, China; 6 Hebei Key Laboratory of Brain Science and Psychiatric-Psychologic Disease, Shijiazhuang 075000, China; 7 Hebei International Joint Research Center for Brain Science, Shijiazhuang 075000, China

**Keywords:** miRNA-132, GSK-3β, tau hyperphosphorylation, diabetic encephalopathy, Alzheimer’s disease

## Abstract

Diabetic encephalopathy (DE) is a global concern and Gordian knot worldwide. miRNA-132 (miR-132) is a class of negative gene regulators that promote diabetic pathologic mechanisms and its complications. However, the molecular mechanisms of miR-132 in DE are elusive, thus an alternative therapeutic strategy is urgently in demand. The present study explored the protective effect and the underlying mechanism of miR-132 on DE via the GSK-β/Tau signaling pathway. Experimentally, a type 2 DM rat model was developed by incorporating a high-fat diet and streptozotocin injection. Further, the DE model was screened via the Morris Water Maze test. Primary hippocampal neurons and HT-22 cells were used for *in vitro* analysis. We found that hyperglycemia exacerbates cognitive impairment in T2DM rats. When we isolated the primary hippocampus neurons, the expression of miR-132 RNA was low in both the DE hippocampus and primary neurons. GSK-3β and Tau 404 were highly expressed in injured HT-22 cells and diabetic hippocampal tissues. miR-132 downregulated the expression of GSK-3β. Besides, a binding and colocalized relationship between GSK3β and Tau was also reported. These findings suggest that miR-132 exerts protective effects from DE injury by repressing GSK-3β expression and alleviating Tau hyperphosphorylation in HT-22 cells and hippocampus tissues.

## INTRODUCTION

Diabetes is a heterogeneous mix of health conditions characterized by glucose dysregulation. According to the Heart Disease and Stroke Statistics-2019 Update, a report from the American Heart Association, nearly 26, 9.4, and 91.8 million adults have diagnosed diabetes, undiagnosed diabetes, and prediabetes in the U.S., respectively. In 2017, the cost of DM was estimated at $327 billion, up by 26 % from 2012 [1]. Following a report by Cho et al., 451 million people had diabetes in 2017 globally, and the number is expected to rise to nearly 693 million by 2045 [2]. People with diabetes have an increased risk of developing several life-threatening health conditions, such as cardiovascular diseases [3], stroke [4], encephalopathy [5], nephropathy [6], cancer [7], oculopathy [8], and neuropathy [9], resulting in higher medical care costs.

Diabetic encephalopathy (DE) is characterized by the development of cognitive dysfunction, paresthesia, numbness, burning, and neuropsychiatric disabilities [10–12]. Diabetic patients with a diagnosis of fewer than 12 months have a 10% possibility to suffer from diabetic neuropathy, which increases to as high as 50% at 25 years after diagnosis [13–16]. However, a majority of patients are, in most cases, diagnosed at the middle/advanced stage, thus fail to receive a good outcome. Due to this, novel theranostic targets should urgently be explored to improve the therapeutic effects and outcomes of DE.

MicroRNAs (miRs), firstly discovered in 2001 [17], are single-stranded RNA of 21-23 nucleotides in length. miRNAs are a class of negative gene regulators that modulate pathologic mechanisms occurring in diabetes and associated complications [18, 19]. So far, miR sequences from 271 organisms, 38,589 hairpin precursors, and 48,860 mature miRNAs have been annotated in mirBase [20]. In general, a single miRNA may target multiple genes, and vice versa, leading to a potentially complicated miRNA-mediated signaling network. Recent studies have demonstrated that miRs play important roles in cardiovascular diseases [21], stroke [22], cancer [23, 24], diabetes [25], among other complications [26]. Of note, dysregulation of miRs contributes to the development and progression of DE.

Glycogen synthase kinase-3β (GSK-3β), firstly isolated from rabbit skeletal muscle, is one of the few protein kinases that are inactivated via phosphorylation [27] (Supplementary Figure 1), it phosphorylates glycogen synthase involved in blood glucose regulation. Notably, the high expression of GSK-3β is correlated with insulin resistance and insulin deficiency. On the other hand, Alzheimer’s disease (AD), a neurodegenerative disease that is pathologically characterized by typically senile plaques (SPs) formed from Aβ deposition, and neurofibrillary tangles (NFTs) composed of tau hyperphosphorylation [28]. GSK-3β plays an important role in the hyperphosphorylation of microtubule-associated protein Tau, one of the pathological features in AD. Based on a recent literature report, GSK-3β is potentially involved in the pathogenesis of DE. This has shifted much attention from researchers to explore the association of GSK-3β with the occurrence of DM [28]. In the present work, we explored whether miR-132 potentially downregulates the expression of GSK-3β in injured HT-22 cells.

## RESULTS

### Hyperglycemia exacerbates the cognitive damage in T2DM rats

During the diabetes modeling process, the diabetic group was highly presented with polyphagia, polydispia, polyuria, weariness, emaciation, and sallow hair, compared to the control group. Notably, 3 rats were eliminated on the 3^rd^ day because their blood glucose level was less than 16.7 mM. Also, 5 diabetic rats succumbed to hyperglycemia.

**Figure 1:**
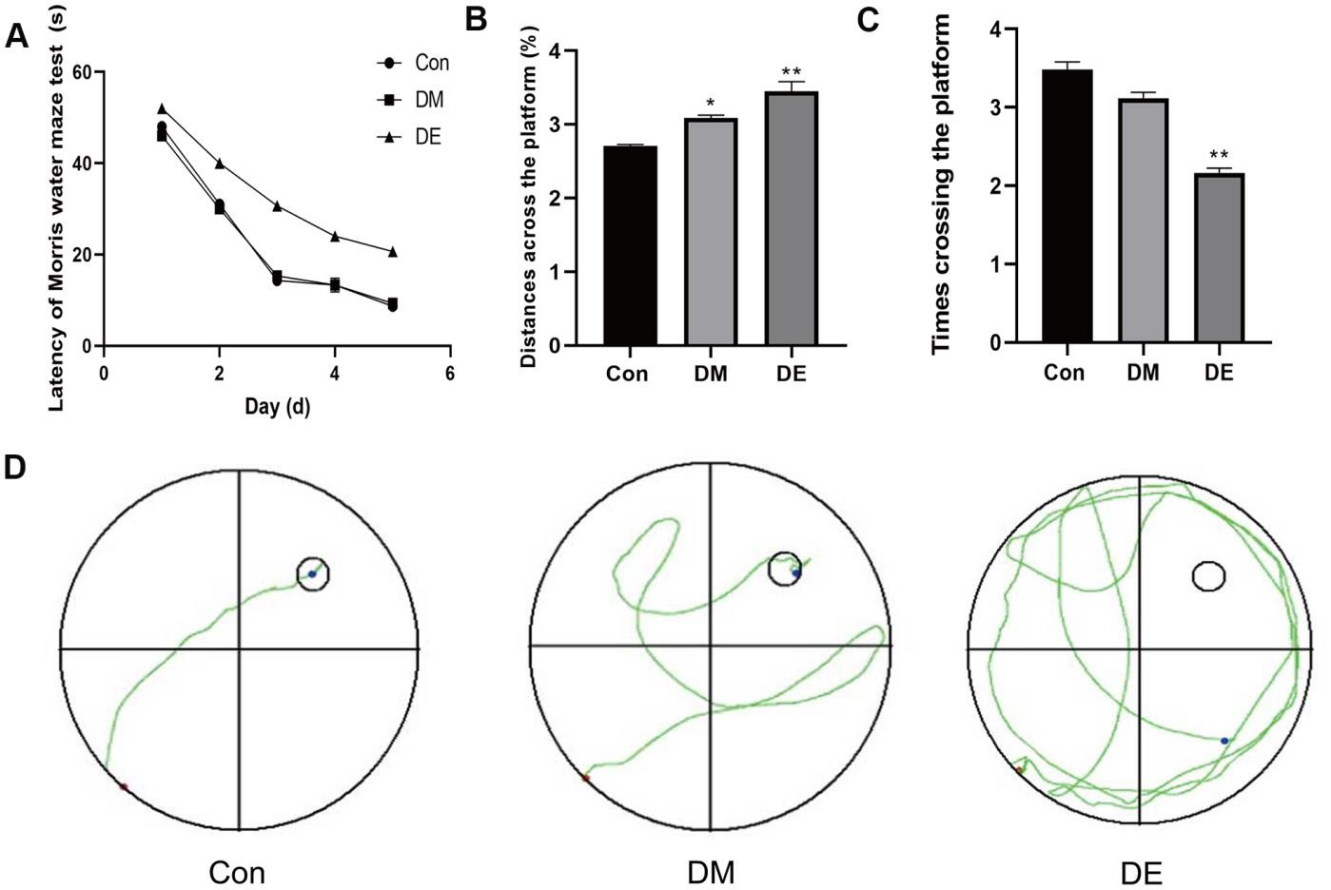
**Hyperglycemia exacerbates the cognitive damage in T2DM rats.** (**A**) The escape latency (EL) of the Morris water maze of three groups is not significantly different on the 1^st^-2^nd^ day (P > 0.05). From the 3^rd^ to 5^th^ day, there is a significant difference of EL between the DE and CG groups, as well as the DM group (*P* < 0.05). In the entire process, there is no significant difference between the DM and CG groups. (**B**) There is a significant difference in the distances across the platform between CG and DM groups, as well as the DE group (*P* < 0.05), whereas no significant difference is evident between the DM group and the DE group. (**C**) The number of rats crossing the platform among the three groups shows a decreasing trend, with no statistical significance between Con and DM (*P* > 0.05), but there is a significance between Con and DE (*P* < 0.05). (**D**) The Morris water maze tests show a confusing trajectory in the DE group, which makes it difficult for rats to find the platform. However, the trajectory of the CG and the DM group is clear and they both directly travel to the platform. Data are presented as the mean ± SD (n = 3 per group). * P < 0.01 vs. control group, ** P < 0.05 vs. the control group, n = 5 for each group.

The escape latency (EL) of the Morris water maze of 3 groups were not significantly different on the 1^st^-2^nd^ day (*P* > 0.05). From the 3^rd^ to 5^th^ day, there was a significant difference in EL between the DE group and the control group (CG), as well as the DM group (*P* < 0.05). During the whole process, no significant difference was reported between the DM and CG groups (Figure 1A).

Besides, there was a significant difference in the distances across the platform between CG and DM, as well as the DE group (*P* < 0.05), whereas no significant difference was reported between the DM group and the DE group (*P* > 0.05, Figure 1B). The number of rats crossing the platform among the three groups showed a decreasing trend, with no statistical significance (*P* > 0.05, Figure 1C). The Morris water maze tests showed a confusing trajectory in the DE group, suggesting the rats could hardly find the platform. However, the trajectory of the CG and the DM groups was clear, whereby the rats directly migrated to the platform (Figure 1D).

### miR-132 is expressed in low levels in DE rat hippocampal tissues and injured HT-22 cells

**Figure 2:**
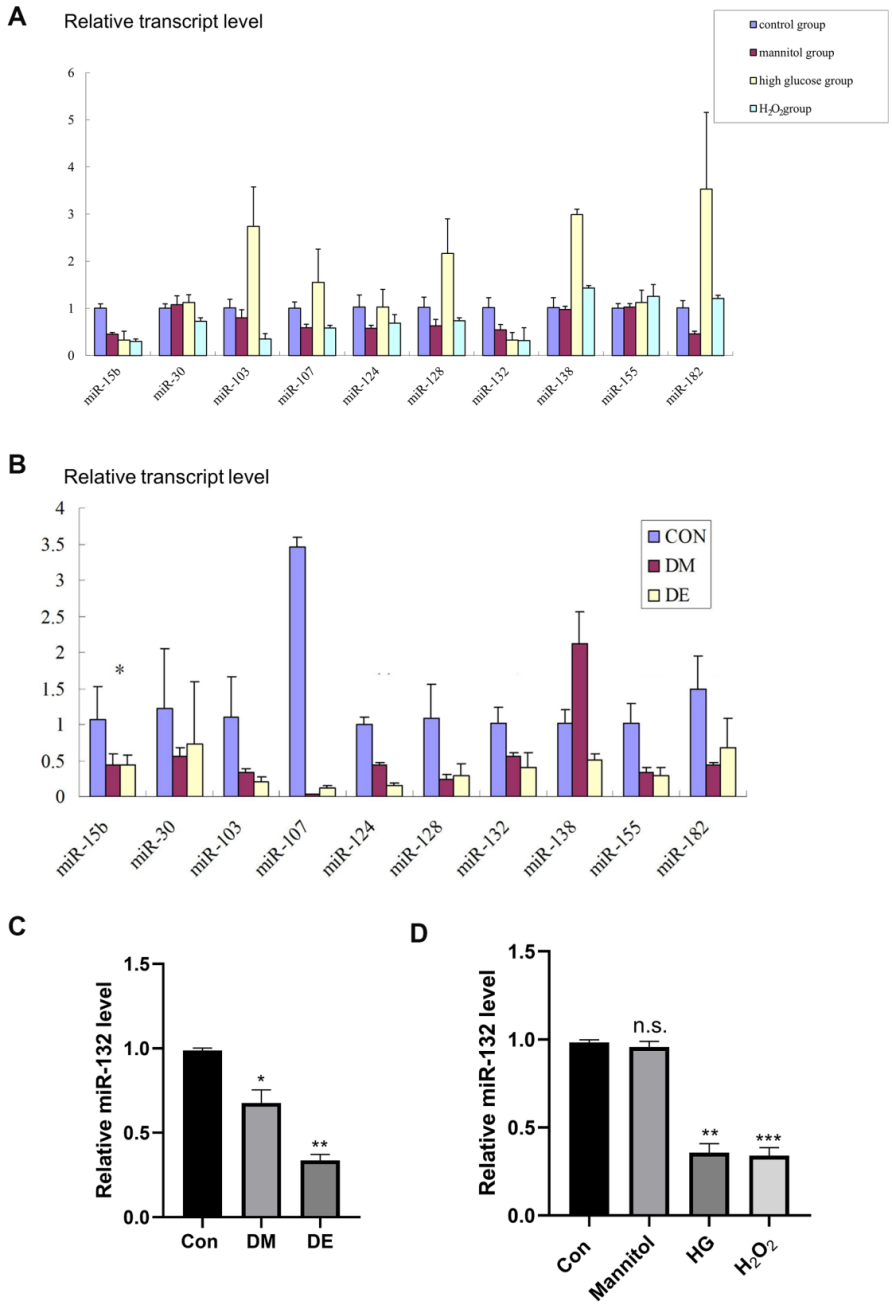
**Low miR-132 expression in DE rat hippocampal tissues and injured HT-22 cells.** (**A**) The expression levels of 10 different miRs in primary hippocampal neurons based on the qRT-PCR analysis. (**B**) The expression levels of 10 different miRs in hippocampal tissues based on the qRT-PCR analysis. *, **, and *** means *P* < 0.05, < 0.01, < 0.001 vs Con; n =3 for each group. (**C**) There is a significant difference of miR-132 between the CG and DM groups, as well as the DE group in hippocampal tissues (*P* < 0.01). (**D**) The relative expression levels of miR-132 are lower in primary hippocampal neurons treated with mannitol, high glucose, and H_2_O_2_ compared to the control group. Data are presented as the mean ± SD (n = 3 per group). * *P* < 0.01 vs. control group, ** *P* < 0.05 vs. the control group, n =3 for each group.

Here, 10 miRNAs related to brain-derived neurotrophic factor (BDNF) and silent information regulator 1 (SIRT1) were selected as potential AD-related miRNAs, according to the instructions of TargetScanHuman 7.0 as previously reported [29, 30]. They included: miR-132, miR-30c, miR -138, miR-124, miR-128, miR-155, miR-182, miR -103, miR -107, and miR -15b (Figure 2A, 2B and Supplementary Table 1). Of note, oxidative stress is a typical pathological alteration of diabetic encephalopathy [12]. BDNF [31] and silent information regulator 1 (SIRT1) [12] are the two known molecules associated with oxidative stress. Therefore, in our experiment, we chose the miRNAs that target BDNF and SIRT1.

We found a significant difference in miR-132 expression between the CG and DM groups, as well as the DE group in hippocampal tissues (*P* < 0.01, Figure 2C). Besides, the relative expression levels of miR-132 were lower in primary hippocampal neurons treated with mannitol, high glucose, and H_2_O_2_ compared to the control group (*P* < 0.01, Figure 2D).

### GSK-3β and Tau404 are highly expressed in injured HT-22 cells and diabetic hippocampal tissues

**Figure 3:**
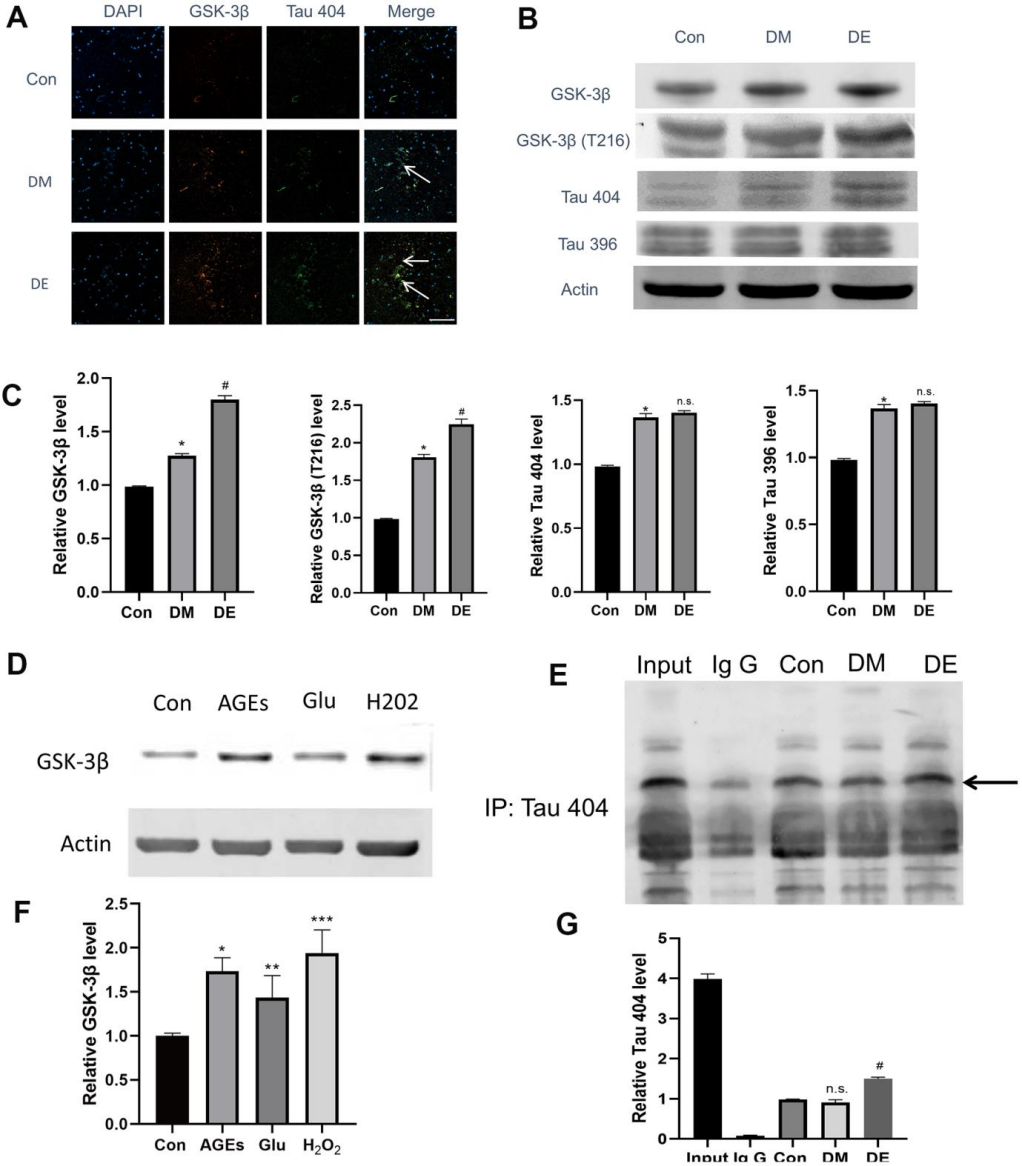
**High expression of GSK-3β and Tau404 in injured HT-22 cells and diabetic hippocampal tissues.** (**A**) Immunostaining analysis showing the enhanced colocalized relationship in Con, DM, and DE groups, progressively (as evidenced by overplay yellow areas in Merge image). Also, GSK-3β and Tau 404 Con are highly expressed in DM, and DE groups, progressively. (**B**, **C**) The immunoblot suggesting a high expression of GSK-3β, GSK-3β(T216), and Tau 404 in Con, DM, and DE groups, progressively. However, the expression levels of Tau 396 and Tau 404 are not different between DM and DE groups. (**D**, **F**) *In vitro* experiment (mimicking) which demonstrates higher expression levels of GSK-3β in Glu, AGEs, and H_2_O_2_ groups, vs Con in HT-22 cells. (**E**, **G**) The IP analysis demonstrating a binding relationship between GSK-3β and Tau 404, and this relationship is enhanced in DM and DE groups. *P < 0.05 vs. control group, ^#^*P* < 0.05 vs. the DM group, n =3 for each group. For Figure 3F, *, **, and *** means *P* < 0.05, < 0.01, < 0.001 vs Con; bar = 100μm.

We firstly explored whether there was a binding and colocalized relationship between GSK-3β and Tau 404. Immunostaining results demonstrated that the colocalized relationship was enhanced in Con, DM, and DE groups, progressively (as evidenced by the overplay yellow areas in Merge image, Figure 3A). The IP results showed that there is a binding relationship between GSK-3βand Tau 404, notably, this relationship was enhanced in DM and DE groups (Figure 3E, 3G). Furthermore, GSK-3β and Tau 404 were highly expressed in Con, DM, and DE groups, progressively (Figure 3A, Supplementary Figure 2). Following immunoblot analysis, the expressions of GSK-3β, GSK-3β(T216), and Tau 404 were high in Con, DM, and DE groups, progressively (Figure 3B, 3C). However, the expression levels of Tau 396 and Tau 404 were similar between DM and DE groups (Figure 3B, 3C). *In vitro* analysis (mimicking) also demonstrated that the expression levels of GSK-3β were higher in Glu, AGEs, and H_2_O_2_ groups, than that of Con in HT-22 cells (Figure 3D, 3F).

### miR-132 downregulates the *in vitro* expression of GSK-3β in injured HT-22 cells

**Figure 4:**
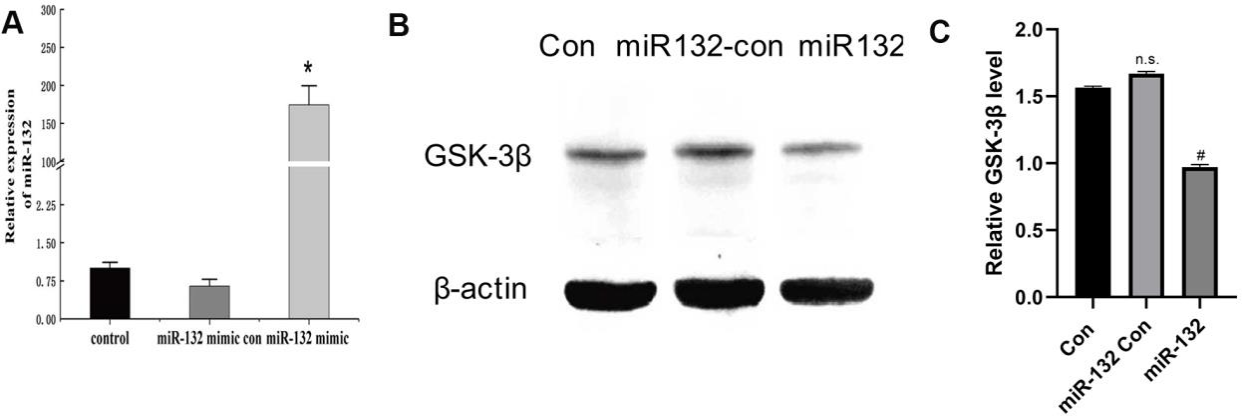
**Successful transfection of synthetic miR-132 mimics, miR-con, and miR-132 were d into HT-22 cells.** (**A**) The transfection of miR-132 is was successfully constructed in HT-22 cells. (**B**) The Western blot analysis showing that miR-132 downregulates the expression of GSK-3β. (**C**) The statistical figure of Figure 4B. *P < 0.01 vs. control group, ^#^*P* < 0.05 vs. the miR-132 Con group, n = 3 for each group.

**Figure 5:**
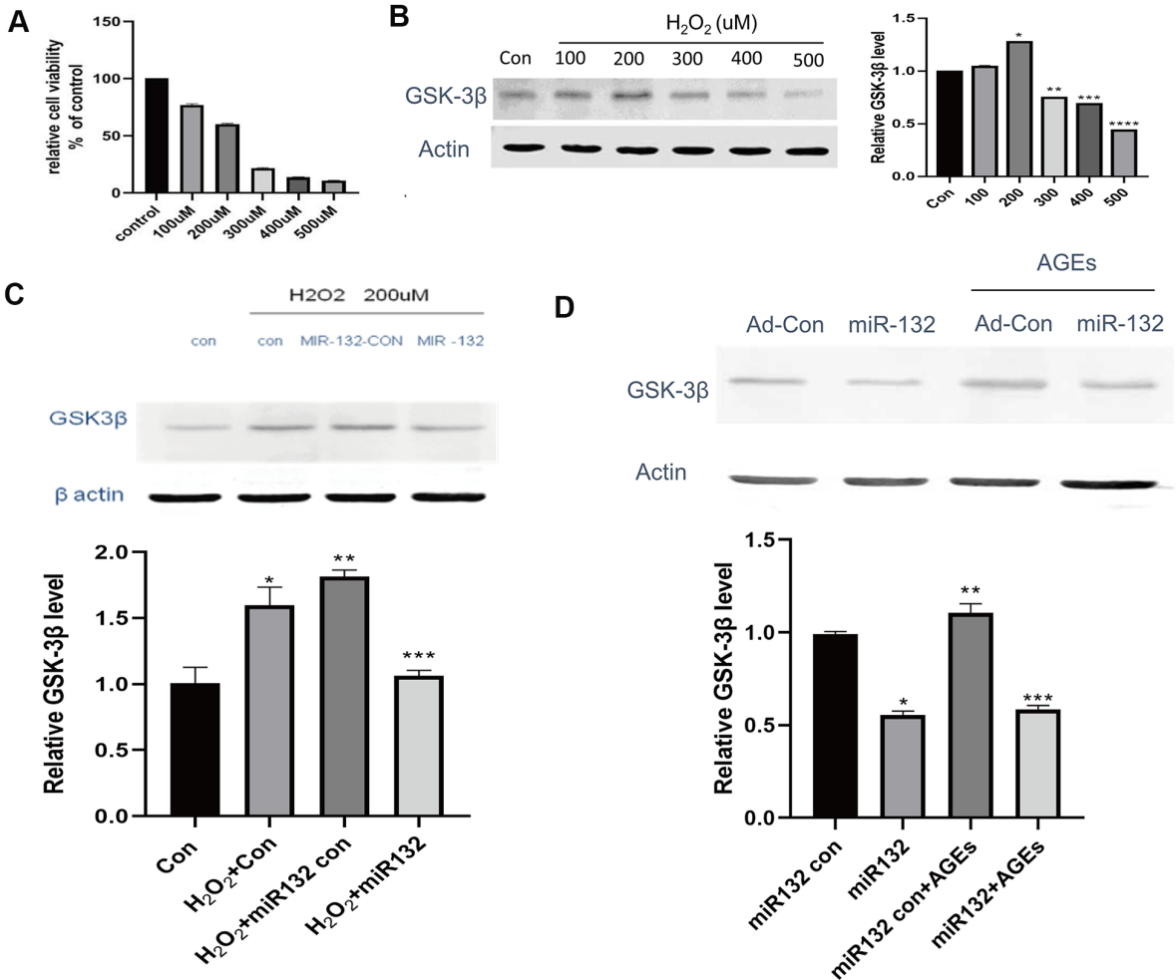
**miR-132 downregulates the expression of GSK-3β and GSK-3β in injured HT-22 cells *in vitro*.** (**A**) The relative cell viability is nearly 60% under 200 μM H_2_O_2_, which contributes to the cellular stress state. (**B**) The expression levels of GSK-3β among 5 groups of different concentrations H_2_O_2_. (**C**) miR-132 potentially reverses the upregulated levels of GSK-3β under H_2_O_2_ treatment. (**D**) miR-132 potentially reverses the upregulated levels of GSK-3β under the AGEs treatment. *, **, and *** means *P* < 0.05, < 0.01, < 0.001 vs Con, n = 3 for each group.

In this experiment, synthetic miR-132 mimics, miR-con, and miR-132. were transfected into HT-22 cells (Figure 4A). Compared to the CG and miR-132 mimics, the expression level of GSK-3β was lower (*P* < 0.05, Figure 4B, 4C). Then, we optimized the concentration of H_2_O_2_ and AGEs in HT-22 cells and found that the relative cell viability was nearly 60% under 200 μM H_2_O_2_, which contributed to the cellular stress state [32] (Figure 5A). Moreover, the expression levels of GSK-3β was highest under treatment of 200 μM H_2_O_2_ (Figure 5B). miR-132 could reverse the upregulated levels of GSK-3β under the treatment of H_2_O_2_ and AGEs (Figure 5C, 5D).

## DISCUSSION

Diabetic encephalopathy is characterized by the development of various conditions, including cognitive dysfunction, paresthesia, numbness, burning, and neuropsychiatric disabilities [10, 11]. Previous studies confirmed that DM patients are at high risk of developing cognitive decline in the clinic [33]. Moreover, experimental literature reports also suggested that cognitive decline exists in diabetic rats [34]. The integrity of hippocampal neurons especially the axon plays a pivotal role in DM-related cognitive damage [35]. Herein, we found that that high-fat diet and STZ-induced hyperglycemia is closely correlated with cognitive decline.

MicroRNAs are much too short to code for protein and instead play significant roles in regulating gene expression. In humans, they regulate most protein-coding genes, including genes important in diabetes and other diseases [36, 37]. In another study, Esguerra reported that the expression of miR-132 is less abundant in T2DM islets [37]. Elsewhere, Zhou and colleagues enrolled 118 gestational diabetes mellitus (GDM) women and collected their blood samples. They reported reduced expression of miR-132 in serum and placenta tissues of GDM, suggesting that serum miR-132 is a potential candidate biomarker in the diagnosis of GDM. Also, miR-132 potentially offered protection from GDM by enhancing the trophoblastic cell proliferation [36]. Additionally, Shang et al. found that overexpression of miR-132 significantly enhanced glucose-stimulated insulin secretion in insular 832/3 and 832/13 cells, and restored insulin responses to glucagon-like peptide 1 (GLP-1) in 832/13 cells [38]. However, there is no current literature report on the roles of miR-132 in DE. In our study, through an *in vitro* experiment, we discovered that miR-132 RNA expression is low in DM and DE rats, as well as the injured HT-22 cell model (Figure 2B), suggesting the potential theranostic value of miR-132 in DE.

Alzheimer’s disease (AD), also regarded as Type 3 diabetes (T3D), is closely related to DM [28, 39]. In AD, GSK-3β plays an important role in the hyperphosphorylation of the microtubule-associated protein tau, which is one of the pathological features in AD. Similarly, in DM, GSK-3β is the crucial enzyme of glycogen synthesis, thereby playing a key role in blood glucose regulation. More importantly, GSK-3β is one of the key factors contributing to insulin deficiency and insulin resistance. Notably, insulin resistance is an important hallmark of the occurrence and development of DM [28]. In this study, we demonstrated that GSK-3β is highly expressed in injured HT-22 cells and diabetic hippocampal tissues, compared to the controls. However, the overexpression of GSK-3β is positively associated with tau hyperphosphorylation.

Recently, El Fatimy’s reported that miR-132 provides neuroprotection for tauopathies via multiple signaling pathways [40]. However, because their single experiment could not provide satisfactory evidence for the scientific community, we performed additional experiments for further validation of the previous findings. First, we used immunoprecipitation to directly confirm whether there is a binding relationship between GSK-3β and Tau (phosphorylation site of Tau). Second, we performed an immunofluorescence experiment to explore the colocalized relationship of GSK-3β with Tau 404. Third, since El Fatimy’s work did not use 404 phosphorylation sites of Tau, we, therefore, assessed the role of Tau 404 and its relationship with GSK-3β in DE tissues.

Tau is a microtubule-associated protein that plays a role in stabilizing neuronal microtubules, thereby promoting axonal outgrowth. Structurally, tau is a natively unfolded protein, which is highly soluble and exhibits little tendency for aggregation [41, 42]. Hyperphosphorylation and aggregation of the microtubule-associated protein tau in the brain are pathological hallmarks of a large family of neurodegenerative disorders, called tauopathies, which include Alzheimer's disease and DE [43]. Tau hyperphosphorylation is regulated by tau kinases including GSK-3β [34, 44]. Indeed, the present findings demonstrated that Tau hyperphosphorylation is higher in injured HT-22 cells and diabetic hippocampal tissues compared to the controls. Also, the overexpression of Tau 404 was associated with tau hyperphosphorylation.

Of note, DE is a global concern a Gordian knot worldwide [13]. Currently, pharmacological therapies have shown no success in preventing DE-associated diabetes. This calls for an urgent search for an alternative therapeutic strategy [35]. In the present study, we revealed that exogenous transfection of miR-132 may downregulate the expression of GSK-3β in injured HT-22 cells *in vitro*. Combining immunofluorescence techniques and molecular analyses *in vivo* and in *vitro*, the present study provides the first time report on protective roles of miR-132 against diabetic encephalopathy injury by repressing GSK-3β expression and alleviating Tau hyperphosphorylation. Firstly, we successfully constructed the DE model using the STZ methods and discovered that hyperglycemia exacerbates the cognitive damage in T2DM rats, as previously reported [34]. Secondly, we isolated primary hippocampus neurons and found that expression of miR-132 RNA is low in both the DE hippocampus and primary neurons. Third, GSK-3β and Tau404 were highly expressed in injured HT-22 cells and diabetic hippocampal tissues, whereas miR-132 downregulated the expression of GSK-3β in injured HT-22 cells. Overall, these findings propose miR-132 as a new therapeutic target to alleviate the DE-induced cognitive decline, which is helpful for the clinical drug design in the management of DE and AD.

## MATERIALS AND METHODS

### Experimental animal and DE model

Male adult (6-8 weeks) and neonatal (1 d) Sprague Dawley (SD) rats were purchased from the Animal Experimental Center of the Hebei Medical University. Animal experiments were approved by the Instructional Animal Care and Use Committee of Hebei Medical University. Adult SD rats were fed in Animal Lab, First Affiliated Hospital of Hebei Medical University with adequate food and water under a 12-h dark-light cycle, and a temperature (23° C) and humidity (50 %) controlled room. All procedures were approved by the Animal Ethical Experimentation Committee of Hebei Medical University.

Notably, 40 rats were fed on a high-fat diet (containing 35% fat) for 4 weeks and assigned randomly to the experimental group (n=30) and control group (n=10). Streptozocin (STZ, 30 mg/kg, i.p.) and citrate buffer solution (30 mg/kg, i.p.) was administered in the experimental and control groups, respectively a 12 h fasting. Then, all rats were fed on free food and water for 72 h, after which the experimental rats were made to fast for 12 h because the model could only be successful if the caudal vein blood glucose were more than 16.7 mmo1/L (Table 1, 2).

**Table 1:** The average body weight of diabetic mice.

	**Diabetic group**	**Normal group**	**T value**	***P***
**4 weeks**	362.38±64.00	366.00±84.3	−0.132	0.896
**8 weeks**	390.24±67.98	458.50±95.98	−2.285	0.03
**12 weeks**	405.24±63.92	535.50±93.76	−4.552	0.000
**16 weeks**	417.62±14.02	598.00±84.86	−6.585	0.000
**20 weeks**	420.00±75.37	658.50±78.14	−8.14	0.000
**23 weeks**	413.80±63.99	718.00±72.12	−11.884	0.000

**Table 2:** Average of blood glucose concentration (mg/dl).

	**Diabetic group**	**Normal group**	**T value**	**P**
**4 weeks**	334.27±70.96	122.22±12.66	13.26	0.000
**8 weeks**	312.34±49.81	123.12±15.06	15.94	0.000
**12 weeks**	331.80±35.76	129.06±20.49	16.59	0.000
**16 weeks**	305.82±59.72	129.96±19.06	12.25	0.000
**20 weeks**	324.60±44.33	131.22±15.77	17.77	0.000
**23 weeks**	314.83±52.33	132.30±15.35	10.73	0.000

### Morris water maze

Morris water maze test was performed to evaluate the cognitive function as previously reported but with minor changes, for example, we used the method that could distinguish the DE group from the DM group [35]. In this experiment, rats injected with STZ after 22 weeks were randomly placed inside the pool at 4 possible start locations. Each rat was conditioned three times per day to let them adapt to the pool environment for 5 days, then allowed up to 5 s to locate the platform. The trial was terminated whenever the rat found the platform within 60 s. The latency was 60 s if the rats failed to find the platform within 60 s. Behaviors of rats were tracked, and we evaluated the escape latency. The latency, distance, and swimming speed were recorded using an automated video tracking software package (NoldusEtho Vision 2.3.19, Netherland).

### Antibodies and reagents

Rabbit anti-β-actin, S9-GSK-3β, T Tau 404 (1:1000), MAP-2 (1:100), mouse anti- S9-GSK-3β, and HRP-linked secondary antibodies (1:5000), FITC-linked secondary antibody (1:100) were purchased from Cell Signaling Technology (Danvers, USA). Rabbit anti-216-GSK-3β and Tau 396 were purchased from Absin (Shanghai, China).

STZ was purchased from Enzo Biochem (New York, USA). High glucose Dulbecco’s modified Eagle’s medium (DMEM), Neurobasal-A medium, fetal bovine serum (FBS), trypsin, Penicillin-Streptomycin-Glutamine, B-27 Supplement (50X) were purchased from Gibco (Waltham, USA). Poly-L-lysine was purchased from Sigma (USA). Triton X-100 was obtained from Bio-High Technology (Shijiazhuang, China).

### Cell culture and treatment

We purchased the HT-22 cells from American Type Culture Collection (ATCC, USA). The HT-22 cells are exposed to mannitol, HG, and H_2_O_2_. The mannitol group was the osmotic control for HG, whereas the H_2_O_2_ was used to generate more reactive oxygen species [45, 46]. Neonatal rat neurons were isolated from 1-day-old SD rats. The isolation of primary hippocampal neurons was performed as previously described [40, 47–49]. Briefly, neonatal SD rats were sterilized with alcohol and sacrificed for brain tissues. The tissues were soaked in 4° C D-Hanks liquid and the hippocampus was isolated using microscope forceps under a microscope. We peeled the pia mater and blood vessels on ice. The peeled hippocampal tissues were digested by pancreatin, centrifuged, inoculated, and purified. First, we obtained the hippocampal tissues then neonatal rat hippocampal neurons as the final isolation.

To identify the primary hippocampal neurons, we used a previously described protocol but with minor modifications [50, 51]: Onto the medium, we added 3.7% paraformaldehyde and left to stand for 10min at room temperature (RT), followed by three times PBS wash, each for 5min; 2). Then, 0.1% TritonX-100 was added, left to stand for 5 min at RT, and washed in PBS thrice each for 5min at RT. Subsequently, cells were incubated with 3% Bovine Serum Albumin (BSA) confining liquid for 30min at RT, then again incubated with Rabbit anti-MAP-2 antibody (1:100) at 4° C overnight and washed in PBS thrice, each for 5 min. Further, cells were incubated with FITC-linked secondary antibody (1:100) for 30 min at RT and washed in PBS thrice, each for 5min. Then, we mounted cells using DAPI and visualized under a fluorescence microscope.

On experiment day 8, onto the primary hippocampal neurons, 200 mmol/L glucose and 100 mmol/L and mannitol were added and incubated for 24 h. This was to mimic the neuron damage *in vitro*. Then, cells were harvested for further analysis.

### Immunofluorescence and immunochemistry analyses

Hippocampus was harvested, embedded in optimal cutting temperature compound, snap-frozen in liquid nitrogen-cooled isopentane, and sliced into 20-μm-thick sections using a freezing microtome. Then, we fixed the hippocampus sections with 4% paraformaldehyde and blocked with 10% serum in PBS. The sections were incubated with the Rabbit anti-GSK-3β and secondary fluorescent antibody and visualized using a confocal fluorescence microscope (Olympus Corporation, Tokyo, Japan).

### Western blot and immunoprecipitation (IP) analyses

The main operating methods followed previous experiments with minor variations [52, 53] using primary antibodies listed in Section 2.2. Briefly, frozen animal heart tissues were homogenized in ice-cold riPa lysis buffer. The extracts were centrifuged for 20 min at 12,000 x g at 4° C to collect supernatants. Protein lysates (25 μg/lane) were separated in 10% SDS-PAGE and transferred onto PVDF membranes. Membranes were blocked with 7% non-fat milk for 90 min at RT and incubated with the following primary antibodies at 4° C overnight. Thereafter, membranes were incubated with an HRP-linked secondary antibody. We shipped the membranes to Quantity One 4.52 (Bio-rad Laboratories, CA, USA) for analysis. Immunoprecipitation was performed using a kit according to the manufacturer's protocol (Invitrogen, CA, USA).

### miRNA transfection

miR-132 mimics (uaacagucuacagccauggucg, from 5’ to 3’), miRNA mimic negative control (miR-con), were purchased from RiboBio Co., LTD (Guangzhou, China). Synthetic miR-132 mimics, miR-con, were transfected into cells using riboFECTTM CP Transfection Kit (Invitrogen, Waltham, USA) according to the manufacturer’s instructions. Transfection was conducted using riboFECTTM CP Transfection Kit (Invitrogen, Waltham, USA) according to the manufacturer’s instructions.

### Quantitative real-time polymerase chain reaction (qRT-PCR)

Total RNA from primary cultures and mouse hippocampal slice was extracted with TRIzol Reagent (Thermo, Waltham, USA) according to the manufacturer’s instruction. Reverse transcription was performed using PrimeScript RT Master Mix (TaKaRa, Kusatsu, Japan). Quantitative real-time PCR (qRT-PCR) was prepared with the SYBR Select Master Mix (Thermo, Waltham, USA) using Bio-Rad CFX96 (Bio-Rad, CA, USA). We used the housekeeping gene β-actin as an endogenous reference. The relative gene expression values were calculated using the ΔΔCt method.

### Statistical analysis

Data were expressed as mean ± SD. All statistical analyses were performed via GraphPad Prism 8 (San Diego, GraphPad Software Inc, USA), using Student's t-test for comparing two groups or one-way ANOVA. Differences with P values<0.05 were regarded as statistically significant. All experiments were performed in triplicates.

## Supplementary Material

Supplementary Figures

Supplementary Table 1
