# Supplementary material for: Decreased miR-132 plays a crucial role in diabetic encephalopathy by regulating the GSK-3β/Tau pathway
Source: Aging (Albany NY). 2020 Dec 27;13(3):4590–604. doi: 10.18632/aging.202418 (PMC7906212; doi:10.18632/aging.202418)
Supplement: Supplementary Figures [file aging-13-202418-s001.pdf]

SUPPLEMENTARY FIGURES

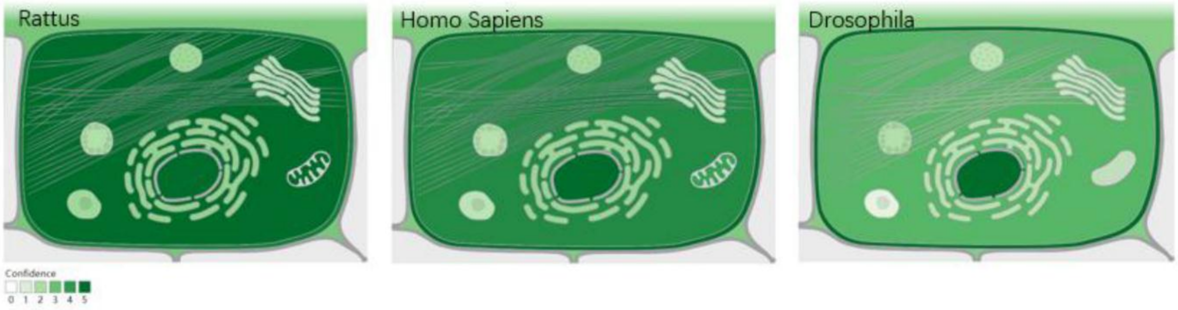

Supplementary Figure 1. Protein expression of GSK-3β in Rattus, Homo Sapiens, and Drosophila.

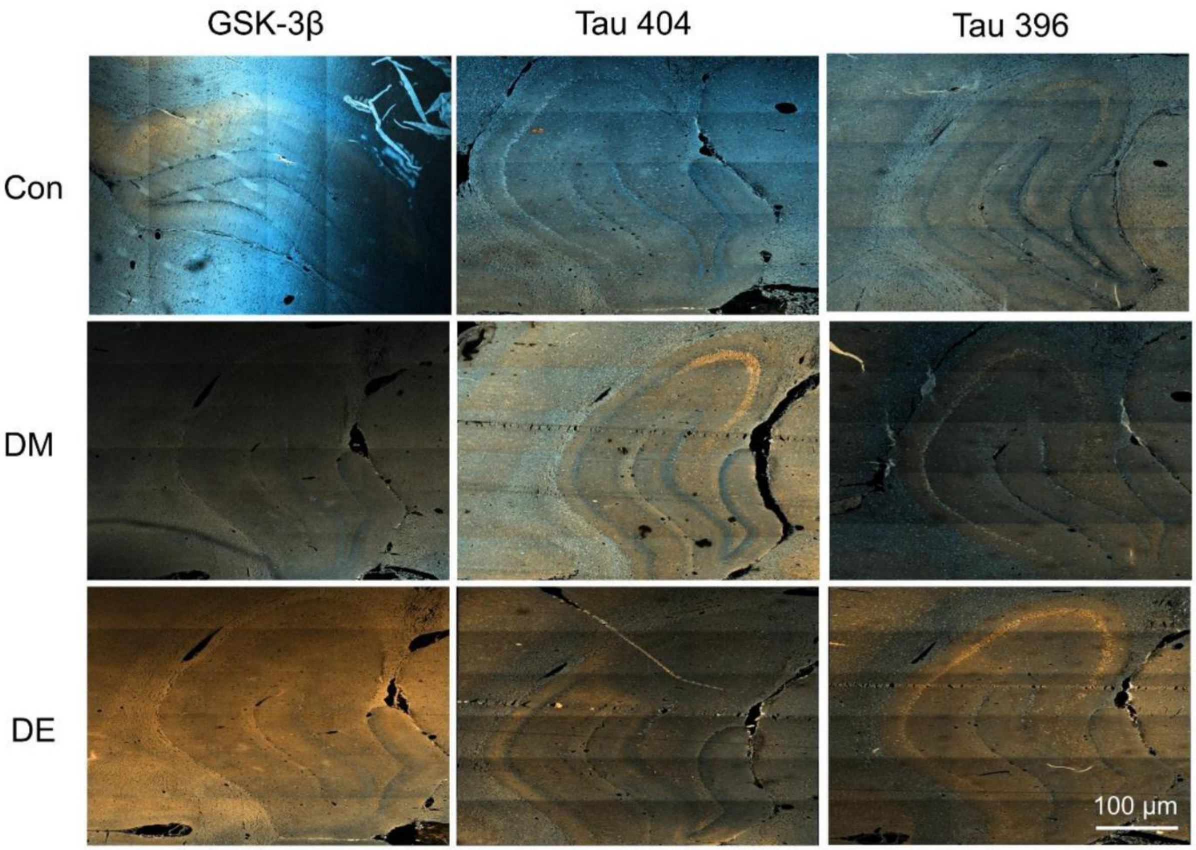

Supplementary Figure 2. The immunochemistry results of GSK-3β, Tau 404, and Tau 396 in Con, DM, and DE groups.
