# Supplementary material for: Decreased miR-132 plays a crucial role in diabetic encephalopathy by regulating the GSK-3β/Tau pathway
Source: Aging (Albany NY). 2020 Dec 27;13(3):4590–604. doi: 10.18632/aging.202418 (PMC7906212; doi:10.18632/aging.202418)
Supplement: Supplementary Table 1 [file aging-13-202418-s002.pdf]

SUPPLEMENTARY TABLE

Supplementary Table 1. Sequence information of miRs.

Gene	Primer (5'-3')
**miR-15b**	UAGCAGCACAUCAUGGUUUACA
**miR-30**	CUGGGAGAGGGUUGUUUACUCC
**miR-103**	AGCAGCAUUGUACAGGGCUAUGA
**miR-107**	AGCUUCUUUACAGUGUUGCCUUGU
**miR-124**	UAAGGCACGCGGUGAAUGCC
**miR-128**	UCACAGUGAACCGGUCUCUUU
**miR-132**	UAACAGUCUACAGCCAUGGUCG
**miR-138**	GCUAUUUCACGACACCAGGGU
**miR-155**	CUCCUACCUGUUAGCAUUAAC
**miR-182**	CGAAUCAUUAUUUGCUGCUCUA
